# The first complete chloroplast genome of *Liparis nervosa* and its phylogenetic position within Orchidaceae

**DOI:** 10.1080/23802359.2019.1681323

**Published:** 2019-10-24

**Authors:** Xujun Wang, Wei Guo, Cuiying Peng, Junsheng Liang, Qidong Liang

**Affiliations:** a Hunan Academy of Forestry, Changsha, P. R. China;; b Taishan Academy of Forestry Sciences, Taian, P. R. China;; c College of Forestry, Central South University of Forestry and Technology, Changsha, P. R. China

**Keywords:** *Liparis nervosa*, chloroplast genome, Illumina sequencing

## Abstract

*Liparis nervosa* is a plant of the family Orchidaceae and mainly distributed in subtropical and tropical regions of the world. In Chinese traditional medicine, it has been used for the treatment of hemostasis, carbuncle, and furuncle for centuries. The chloroplast (cp) genome of *L. nervosa*, sequenced based on next-generation platform (NEOSAT), is 157,274 bp in size. The cp genome encodes 130 genes, including eight rRNA genes, 85 protein-coding genes (PCGs), and 37 tRNA genes. Phylogenetic relationship analysis based on complete cp genome sequences exhibited that both of *L. nervosa* and *L. loeselii* were phylogenetically closer to *Dendrobium officinale*.

*Liparis nervosa* is a species of flowering herbaceous plant of the family Orchidaceae and mainly distributed in subtropical and tropical regions of the world. It grows on rocks and in the shade of trees beside valleys at an altitude of 1000–2100 m (Chen 1999). *Liparis nervosa* and *L. acuminata* look very similar, yet their lip petals have calli of different geometric shapes (Chen 1999). As an extensive used herb in Chinese traditional medicine, *L. nervosa* has been used for the treatment of hemostasis, carbuncle and furuncle. Until recently, most research on *L. nervosa* has focussed on phytochemical constituents (Liu et al. 2016, 2019; Chen et al. 2018). Phenanthrenes have aroused extensive attention for its antitumor activity (Liu et al. 2016). Thus the genomic and genetic knowledge is desperately required to make good use of the germplasm resources of *L. nervosa*. Consequently, in this paper, we reconstructed the chloroplast genome sequences of *L. nervosa*.

The voucher specimen (accession no. DJC_0957_JXQ_Changs) was harvested from an individual *L. nervosa* plant at Dujiachong experimental forest located in Yuhua District, Changsha, Hunan, China (28°06′40″N, 113°01′30″E). It was chilled with dry ice and deposited in a chest freezer at the herbarium of Hunan Academy of Forestry (HAF). The total genomic DNA (gDNA) was isolated from 300 mg of young leaves by CTAB protocol. An Illumina Hiseq 2500 platform was utilised to conduct 2 × 150 bp paired-end sequencing. After trimming, around 5.7 Gb clean data were used to map to the on-line published cp genome of *L. loeselii* (GenBank no. MF374688; Krawczyk et al. 2018) using bowtie2 v2.2.4 (Langmead and Salzberg 2012). Filtered reads were pooled and used for *de novo* assembly. The scaffolds were obtained using SSPACE v2.0 (Bankevich et al. 2012) and patched using Gapfiller v2.1.1 (Boetzer and Pirovano 2012), respectively. The annotation was performed using DOGMA pipeline (Wyman et al. 2004) and BLAST searches, then adjusted manually.

The complete cp genome of *L. nervosa* (GenBank no. MN480463) is a circular DNA molecule with 157,274 bp in size having 36.97% of total GC content. There are 130 genes annotated in the cp genome, containing eight rRNA genes, 85 PCGs and 37 tRNA genes. Eight tRNA genes, seven PCGs, and four rRNA genes were duplicated in the inverted repeat regions (IRs). Two PCGs (*ycf3* and *clpP*) harbour two introns each while six of them (*atpF*, *rpl2*, *rpoC1*, *rps16*, *ndhA*, and *ndhB*), harbour one intron each.

For phylogenetic maximum-likelihood (ML) analysis, multiple alignment was carried using MAFFT v7.2 with 15 published cp genomes downloaded from Genbank (Katoh and Standley 2013). The ML tree, reconstructed using RAxML v8.2.10 (Stamatakis 2014) with 1000 bootstraps under GTR model, showed that both of *L. nervosa* and *L. loeselii* were phylogenetically closer to *Dendrobium officinale* than others (Figure 1), which was consistent with the prior phylogenetic study (Krawczyk et al. 2018; Li et al. 2019; Zhang et al. 2019).

**Figure 1.**
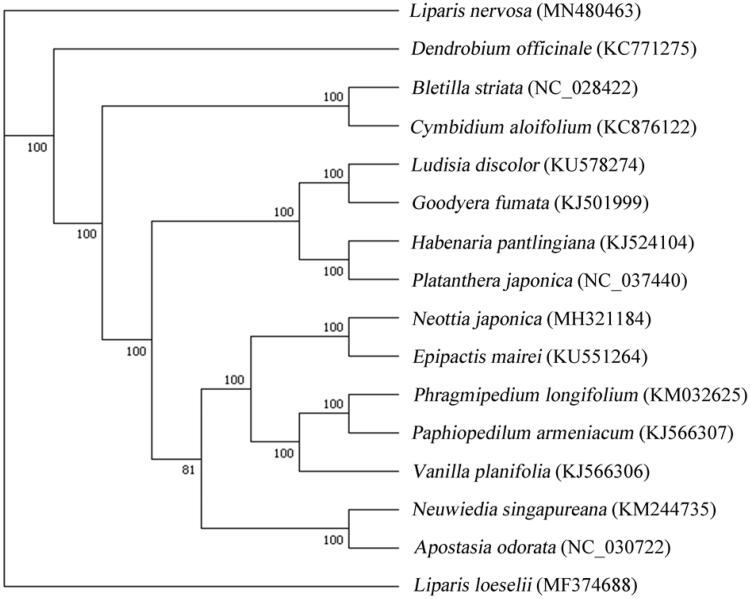
Phylogenetic tree based on 16 complete cp genome sequences. The bootstrap support values are shown next to the branches.
